# Effects of Sugar Cane Molasses Addition on the Fermentation Quality, Microbial Community, and Tastes of Alfalfa Silage

**DOI:** 10.3390/ani11020355

**Published:** 2021-01-31

**Authors:** Runbo Luo, Yangdong Zhang, Fengen Wang, Kaizhen Liu, Guoxin Huang, Nan Zheng, Jiaqi Wang

**Affiliations:** 1 State Key Laboratory of Animal Nutrition, Institute of Animal Science, Chinese Academy of Agricultural Sciences, Beijing 100193, China; lorambo@163.com (R.L.); zhangyangdong@caas.cn (Y.Z.); wfe8520382@163.com (F.W.); 18728187930@163.com (K.L.); huangguoxin1991@163.com (G.H.); zhengnan_1980@126.com (N.Z.); 2 Key Laboratory of Quality & Safety Control for Milk and Dairy Products of Ministry of Agriculture and Rural Affairs, Institute of Animal Sciences, Chinese Academy of Agricultural Sciences, Beijing 100193, China; 3 College of Animal Science, Xinjiang Agriculture University, Urumchi 830091, China

**Keywords:** alfalfa silage, molasses, taste, microbial community, fermentation

## Abstract

**Simple Summary:**

It is difficult for Alfalfa alone to obtain a competitive fermentation quality due to its low content of fermentable carbohydrate and great buffering capacity. Sugar cane molasses additives provide a substrate for the rapid accumulation of lactic acid (LA) and pH reduction while increasing the nutritional quality of silage. The present work aims to study the effects of molasses additives on the fermentation quality and taste evaluation of the alfalfa silage. The microbial communities of the alfalfa silage were also described as the explanation for the changes in silages. The study could give directions on improving the fermentation quality of alfalfa silage and achieve long-term preservation.

**Abstract:**

The objective was to study the effects of sugar cane molasses addition on the fermentation quality and tastes of alfalfa silage. Fresh alfalfa was ensiled with no additive (Control), 1% molasses (M1), 2% molasses (M2), and 3% molasses (M3) for 206 days. The chemical composition and fermentation characteristics of the alfalfa silages were determined, the microbial communities were described by 16S rRNA sequencing, and the tastes were evaluated using an electronic tongue sensing system. With the amount of added molasses (M), most nutrition (dry matter and crude protein) was preserved and water-soluble carbohydrates (WSC) were sufficiently used to promote the fermentation, resulting in a pH reduction from 5.16 to 4.48. The lactic acid (LA) content and LA/acetic acid (AA) significantly increased, indicating that the fermentation had turned to homofermentation. After ensiling, Enterococcus and Lactobacillus were the dominant genus in all treatments and the undesirable microbes were inhibited, resulting in lower propionic acid (PA), butyric acid (BA), and NH3-N production. In addition, bitterness, astringency, and sourness reflected tastes of alfalfa silage, while umami and sourness changed with the amount of added molasses. Therefore, molasses additive had improved the fermentation quality and tastes of alfalfa silage, and the M3 group obtained the ideal pH value (below 4.5) and the best condition for long-term preservation.

## 1. Introduction

Alfalfa is considered a promising high-quality feed for ruminants due to its good palatability and high protein content [1,2], but easily suffers a loss of nutrition caused by aerobic deterioration, especially in humid conditions and rainy climates, and needs an efficient way of preservation. Ensiling is a feasible method to reduce the loss of alfalfa nutrition, improves the feed intake, and obtains long-term preservation, via spontaneous lactic acid (LA) fermentation under anaerobic conditions [3]. With the rapid proliferation of lactic acid bacteria (LAB), the carbohydrates in raw materials are converted into organic acids, mainly LA [4], which lead to a rapid pH reduction, inhibit the growth of harmful microorganisms, and finally obtain a stable condition for forage preservation [5]. During the ensiling process, sufficient fermentable carbohydrates, as the fermentable substrate in the early stage, are crucial for LA production, which reduces the pH value and improves the silage quality [6]. However, it is difficult for alfalfa alone to obtain a competitive fermentation quality due to its insufficiency in fermentable carbohydrate and its great buffering capacity (BC) [7].

Previous studies have suggested that ensiling alfalfa with high-sugar-content forage crops, such as corn [8] and sweet sorghum [9], could significantly improve the fermentation quality of alfalfa silage. Another strategy is to add cheap sources of exogenous sugar additive, such as molasses (M), which has been widely used to accelerate fermentation and improve the quality of alfalfa silage [10]. M additive not only provides sufficient substrates for the rapid accumulation of LA and sharp reduction of pH value, but also increases microbial protein synthesis and increases the nutritional quality [11]. According to a previous study, M additive improved the fermentation quality of alfalfa-mixed silage, and 2.5% M was enough and more suitable than 5.0% M for practical application [12]. On the contrary, other studies have found that M additive increases aerobic stability, with no effects on the fermentation quality of alfalfa silage [13,14]. Therefore, the fermentation quality of alfalfa silage prepared with M additive has not sufficiently been studied yet and remains controversial.

Moreover, the tastes of silage change after ensiling and often improve the palatability [6], but little information is available regarding the tastes of alfalfa silage. The electronic tongue sensing system (e-tongue) is an objective method for taste evaluation, which offers taste results closer to human sensory evaluation. As the working principle of the potentiometry-based e-tongue, the electrode potentials were measured between the outer sensor membrane boundary and reference electrode when the taste substance interacted with the sensor membrane, and the potential change was then converted into taste information though pattern recognition methods; and the basic gustatory feelings were classified and recognized, such as sourness, saltiness, bitterness, and umami. Although the e-tongue has been widely used in the production of foods and beverages [15], in pharmaceutical industries [16], and in environmental and fermentation monitoring [17,18,19], it has not yet been extended into feed processing.

In the present work, the objective was to evaluate the fermentation quality of alfalfa silage prepared with M additive, and to achieve long-term preservation for practical application, including chemical composition, fermentation characteristics, and microbial community. The e-tongue was also used, as the first try on feed processing, to evaluate the tastes of alfalfa silage. We hypothesized that molasses addition could improve fermentation quality and change the tastes of alfalfa silage.

## 2. Materials and Methods

### 2.1. Alfalfa Harvest and Silage Preparation

Alfalfa (adrenalin) was cultivated and harvested in the early bloom stage of the fifth crop period (294.90 g kg^−1^ dry matter (DM)) in October in the cultivated pasture of Mengde Dairy Farm located in Tianjin (39° N, 116° E), China. The fresh alfalfa was cut by hand using a sickle at about 8–10 cm above the ground and then chopped into 2–3 cm with a paper-cutter.

The sugarcane molasses (the brix was 87%) was obtained from the local market and used as the additive for ensiling alfalfa. The chopped alfalfa was mixed and divided into equal portions for four treatments: (1) No M added (Control); (2) M added at 1% of fresh material (FM) (M1); (3) M added at 2% of FM (M2); (4) M added at 3% of FM (M3). Then, 3.3 kg of the forage mixture from each treatment was packed into a 4.9 L polyethylene laboratory silo, and sealed with a screw top and plastic tapes, and then kept at ambient temperature (21–25 °C). Sextuplicate silos for each treatment were opened after ensiling for 206 days.

### 2.2. Fermentation Characteristics

First, 20 g of fresh alfalfa and each silage was homogenized with 130 mL cold deionized water (4 °C) for 60 s in a blender, shaken at 4 °C for 24 h with a shaking table, and then filtered with 4 layers of roving cloth into a beaker and further with quantitative filter paper into a triangular flask. The filtrate was immediately determined for its fermentation characteristics.

The pH value of the filtrate was immediately measured with a glass electrode pH meter (Sartorius PB-10, Goettingen, Germany), using the voltammetry method. LA was determined using a Waters Acquity UPLC system (Waters H-Class, Milford, MA, USA) equipped with a Waters 2489 UV/VIS detector and HSS T3 column (2.1 × 100 mm, 1.8 μm). The analytical conditions were as follows: Oven temperature, 35 °C; mobile phase A, 0.1% phosphoric acid; mobile phase B, methanol; flow rate, 0.2 mL/min; injection volume, 5 μL. Acetic acid (AA), propionic acid (PA), and butyric acid (BA) were determined using an Agilent GC system (Agilent 7890A, USA) equipped with an FID detector and an FFAP column (15 m × 0.32 mm × 0.25 μm). The analytical conditions were as follows: Inlet temperature, 250 °C; carrier gas pressure, He 19.991 kPa; oven temperature, programmed; injection volume, 2 μL.

### 2.3. Chemical Composition

The filtrate was also determined for NH3-N content, using the phenol-sodium hypochlorite method, as described by Broderica [20]. The fresh alfalfa and silage samples were dried in an oven at 65 °C for 48 h to estimate the DM; then, samples were ground to pass a 1 mm screen with a Wiley mill (ZM200, Retsch GmbH) for compositional analysis [21].

Crude protein (CP) was determined using the Kjeldahl method according to AOAC standard procedures [21]. Neutral detergent fiber (NDF) and acid detergent fiber (ADF) were determined using a fiber analyzer (A2000I, Ankom Technology, Macedon, NY, USA), as described by Van Soest [22]. The water-soluble carbohydrates (WSC) content was determined using the anthrone colorimetric method, as described by Owens [23].

### 2.4. DNA Extraction

Microbial DNA from each silage sample was extracted using the HiPure Stool DNA Kits (Magen, Guangzhou, China). A 10 g sample was mixed with 90 mL sterilized saline, filtered with gauze, and then centrifuged to collect the pellet.

### 2.5. PCR Amplification and 16S rRNA Sequencing

The V5–V7 hyper variable regions of 16S rRNA were amplified using the universal primers 799F (CCTAYGGGRBGCA) and 1193R (ACGTCATCCCCACCTTCC). The PCR amplification was performed with KOD Polymerase (Toyobo, Osaka, Japan) at 94 °C (2 min), followed by 30 cycles at 98 °C (10 s), 62 °C (30 s), and 68 °C (30 s), and ended with a final extension step at 68 °C for 5 min.

### 2.6. Silage Tastes

The tastes of the silage filtrates were determined using an e-tongue sensing system (INSENT SA402B, Japan). First, 30 mM KCl and 0.3 mM tartaric acid were used as the reference solution, and umami, saltiness, sourness, bitterness, and astringency were evaluated through the outputs of sensors AAE, CT0, CA0, C00, and AE1, respectively. Sensor outputs were converted to “taste information” with the program in the workstation platform, where “1 unit” was defined as the smallest difference that a person could distinguish.

### 2.7. Statistical Analyses

A completely random design was used in the study. All data of chemical composition and fermentation parameters were analyzed by one-way analysis of variance (ANOVA) using SPSS statistics v. 26.0 (IBM, Armonk, NY, USA). Duncan’s test was employed for multiple comparisons. *p* < 0.05 and *p* < 0.01 indicated significant and extremely significant, respectively. The statistical model was as follows:

Y_ij_ = μ + T_i_ + E_ij_

where Y_ij_ represents the observed dependent variables, μ is the overall mean, T_i_ is the effect of treatment, and E_ij_ is the residual error.

## 3. Results

### 3.1. Chemical Composition and pH Value of the Fresh Alfalfa Before Ensiling

The chemical composition of the fresh alfalfa before ensiling is presented in Table 1. The DM content was 294.90 g/kg FM on average, while the NDF, ADF, CP, and WSC contents were 377.20 g/kg DM, 207.78 g/kg DM, 242.15 g/kg DM, and 103.33 g/kg DM, respectively. The pH values of the filtrates from the fresh alfalfa were also determined, as 5.91 on average.

### 3.2. Chemical Composition of the Alfalfa Silage

As shown in Table 1, M additive had a significant effect on the contents of DM, WSC, NDF, and ADF of the alfalfa silage (*p* < 0.05), but no effect on CP content.

With the amount of added M, the DM content of alfalfa silage slightly increased, and the M2 group and M3 group were 10% and 5.4% higher than that of the control group, respectively. Meanwhile, the WSC content decreased after ensiling and remained stable around 30 g/kg DM. The NDF content decreased after ensiling and remained at 68.27–76.94%. The ADF content remained stable after ensiling and remained at 91.71–101.10%. In terms of the CP content, there was no significant difference among treatments, and they were similar to the value before ensiling.

As a result, most WSC of alfalfa silage was consumed, and a part of the NDF was decomposed during the ensiling process, while DM, CP, and ADF contents remained relatively stable. Among all the treatments, the M3 group had the highest DM and CP contents and the lowest NDF content.

### 3.3. Fermentation Characteristics of the Alfalfa Silage

As shown in Table 2, the M additive had a significant effect on the pH value and the contents of LA, AA, PA, NH3-N, and LA/AA of the alfalfa silage (*p* < 0.05), but no effect on BA content.

The pH value of fresh alfalfa was 5.91, which reduced after ensiling, and significantly decreased from 5.16 to 4.48 with the amount of added M. Meanwhile, compared with the control group, the LA content significantly increased by 19.82%, 21.40%, and 44.17% in the M1, M2, and M3 group, respectively, while the AA content significantly decreased by 2.97%, 25.35%, and 32.44%, respectively. LA/AA was correspondingly increased from 3.30 to 6.84. With the amount of added M, the PA content significantly decreased by 24.78%, 67.83%, and 70.43% in the M1, M2, and M3 group, respectively. The BA content was low and decreased by 30.30%, 51.52%, and 87.88%, while the NH3-N content was also significantly decreased by 24.91%, 47.03%, and 48.25% in the M1, M2, and M3 group, respectively.

As a result, the pH value had a rapid reduction during the ensiling process as expected. Among all the treatments, the M3 group had the lowest pH value and AA, PA, BA, and NH3-N contents, as well as the highest LA content and LA/AA.

### 3.4. Tastes of the Alfalfa Silage

**Figure 1:**
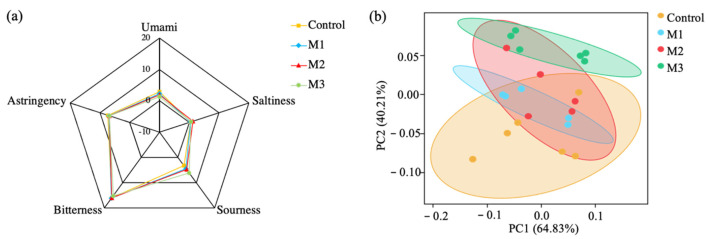
Tastes analysis of the alfalfa silage (*n* = 6). (**a**) Radar charts. (**b**) Principal coordinate analysis.

As presented in Figure 1a, the radar charts display the taste information, with a unit corresponding to the smallest difference in taste that a person can distinguish. All alfalfa silage samples had strong bitterness, astringency, and sourness, which reflected the tastes of alfalfa silage. With the amount of added M, the sourness increased, but the umami decreased, while the bitterness, astringency, and saltiness remained.

Principal coordinate analysis was also performed for the difference in tastes. As shown in Figure 1b, the tastes of the M3 group were clearly distinguished with the control group.

### 3.5. Microbial Community of the Alfalfa Silage

PCR amplification and 16S rRNA sequencing were conducted to systematically describe the microbial communities in the alfalfa silages. As listed in Table 3, the coverage of all samples was above 0.99, indicating most of the bacteria was detected. Compared with the control group samples, lower Chao and Shannon indexes were observed in the M2 and M3 group samples, indicating that the M additive had an obvious influence on silage microorganism. With the amount of added M, lower species were observed in the alfalfa silages.

**Figure 2:**
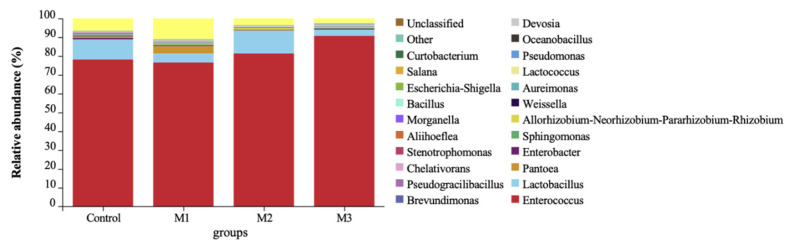
Microbial communities of the alfalfa silages after 206 days of ensiling examined by 16S rRNA sequencing (*n* = 6).

As presented in Figure 2, the dominant genus in alfalfa silages after 206 days of ensiling were Enterococcus and Lactobacillus, accomplished with small amounts of Pantoea, Enterobacter, and Weissella, etc. existing. With the amount of added M, the percentages of dominant genus Enterococcus and Lactobacillus gradually increased and tended to stabilize, which were 88.63% in the control group, 81.33% in the M1 group, 93.20% in the M2 group, and 93.86% in the M3 group.

## 4. Discussion

### 4.1. Chemical Composition of the Alfalfa Silage

A previous study has indicated that DM losses of silage were mainly caused by the proliferation of undesirable microbes such as yeast, mold, and L. buchneri during the ensiling process, and M additive could reduce the DM losses of silage [12]. In the present work, the DM contents remained stable or even slightly increased with the amount of added M, which proved the efficiency of alfalfa nutrition preservation by the ensiling process.

In the ensiling process, WSC played a critical role in silage fermentation and acted as the fermentable substrate in the early stage [24]. It was widely accepted that alfalfa alone was related to a weak fermentability due to its insufficiency of WSC [25]. In the present work, the WSC content of fresh alfalfa was about 30.47 g/kg FM (DM = 294.90 g/kg FM, WSC = 103.33 g/kg DM), and increased in treatments by 33% (M1), 66% (M2), and 98% (M3), respectively. After ensiling, the WSC contents of all treatments decreased to 28.52–37.19 g/kg DM, regardless of the initial contents, which was considered the threshold of WSC for continuing fermentation and pH reduction.

When the pH value was low enough to limit the proteolytic bacteria activities, the protein from the fresh material was preserved [26]. The stable CP contents of the alfalfa silages in the present work proved the efficiency of pH reduction and protein preservation. A lower pH value is related to the acid hydrolysis of more digestible plant cells during the ensilage process [27], and results in more NDF reduction. Meanwhile, the ADF contents of all treatments remained stable after ensiling, which was consistent with previous studies [13].

### 4.2. Fermentation Characteristics of the Alfalfa Silage

pH value is the most significant factor of fermentation quality, which mainly inhibits the proliferation of undesirable microbes and the proteolytic activities [28]. Previous studies have shown that the silage obtained good fermentation quality when the pH value reached 4.5 or below [13], and M additive could affect the final pH value only when the initial WSC content of fresh material was too low [29]. In the present work, the pH value of the alfalfa silage had effectively reduced after ensiling, and M additive had obviously enlarged the reduction. However, only the M3 group obtained the ideal pH values of below 4.5, but in a similar research, alfalfa-mixed silage ensiled with 2% M additive had obtained such ideal pH values [30].

The production of organic acids was mainly responsible for the pH reduction of silage. In these acids, LA is the desirable fermentation product in silage mainly produced by LAB that consumes WSC, whereas AA, PA, and BA are undesirable [31]. AA, considered negatively related to silage Dry Matter intake (DMI) in dairy cows and the voluntary intake when the AA content was more than 20 g/kg DM [32], is mainly produced from the action of heterofermentative LAB, propionibacteria, and enterobacteria, and is also formed from citrate, malate, and amino acid degradation [33]. According to the LAB fermentation pattern, homofermentation mainly produced LA, whereas heterofermentation produced AA with LA [34,35]. In the present work, with the amount of added M, the increase in LA content indicated the sufficient use of WSC and acted as the main reason for pH reduction, while the AA content continued to decrease until below 20 g/kg DM in M2 and M3 groups. In addition, LA/AA was significantly increased with the amount of added M, indicating that the fermentation of alfalfa silage had turned to homofermentation, which was consistent with previous studies [12].

PA is mainly produced by Propionibacterium, as well as Clostridium propionicum and Selenomonas ruminantium [13], which is suppressed in lower pH conditions below 4.5 [36]. BA, which is the result of amino acids fermentation and leads to nutrition losses and is an indicator of bad preservation, is mainly produced by Clostridium butyrate and suppressed in lower pH conditions [37]. HN3-N, the proportion of which was related to proteolysis activity in the ensiling process and negatively related to the voluntary intake of silage [38], was caused by the proliferation of Clostridium spp. and inhibited in lower pH conditions. In the present work, with the amount of added M, the contents of PA and BA decreased and remained at a very low level, which indicated that the Propionibacterium was inhibited by lower pH, and Clostridial fermentation was not developed. Meanwhile, the decline in BA and HN3-N contents indicated a lower CP consumption and a better preservation of the alfalfa silage.

### 4.3. Tastes of the Alfalfa Silage

This is the first time taste evaluation has been conducted on alfalfa silage, and strong bitterness, astringency, and sourness have reflected the tastes of alfalfa silage. Bitterness, produced mainly by some alkaloids and glycosides such as quinine and caffeine, which often exists in the epidermic cells of plants, is often considered distasteful and poisonous to humans [39]. Astringency is produced mainly by tannins and usually considered an appreciated quality. Sourness is generated by organic acids and often reflects the decomposition of nutrition in foodstuffs [15], but in silage, it may be related to the fermentation quality. Meanwhile, umami is produced by some amino acids, especially glutamic acid.

With the amount of added M, the increased sourness indicated that the increase in LA had more contribution on the change in membrane potential than the decrease in AA, PA, and BA contents, which was consistent with the pH values of alfalfa silage. The decreased umami was related to lower protein decomposition.

### 4.4. Microbial Community of the Alfalfa Silage

Enterococcus, Lactobacillus, Pediococcus, and Weissella, etc., as the desirable LABs, existed randomly on the surface of pre-ensiled silage [27]. These LAB strains start the lactic fermentation at the early stage of the ensiling process, and Lactobacillus promotes the pH reduction at the later stage [40]. Enterococci was reported to grow vigorously during fermentation until the pH value declined to below 4.5. In the present work, Enterococcus and Lactobacillus have become the dominant genus in all the alfalfa silage after 206 days of ensiling, which was consistent with former studies [27]. M additive provides additional fermentable substrates for LABs, which promotes domination in the microbial community in the final silage.

Enterobacteria, Clostridial, yeast, and mold were the most undesirable microbes, which may cause aerobic deterioration and result in nutrition losses. Fast acidification and low pH conditions (below 5.0) were critical to the inhibition of their growth [33]. In the present work, Clostridial, yeast, and mold were seldom detected in all the samples, with only a small amount of Enterobacter existing, indicating that these undesirable microbes were suppressed by the dominant genus and obtained a good condition for long-term preservation.

## 5. Conclusions

After ensiling, DM and CP contents of the alfalfa silage were almost unchanged, indicating that most nutrition was preserved. WSC, as the fermentation substrate, was reduced to around 30 g/kg DM, the value of which was considered the threshold for continuing fermentation, and the pH value was also reduced to 5.16. The NDF content decreased mainly due to more plant cell digestion in lower pH conditions, while the ADF content remained stable.

With the amount of added M, the DM, CP, and ADF contents of the alfalfa silages remained stable, while WSC residues were similar around the threshold. pH values decreased significantly from 5.16 to 4.48, and resulted in the reduction of NDF, as well as the increase in LA content and LA/AA, indicating that the fermentation of alfalfa silage had turned to homofermentation. Moreover, Enterococcus and Lactobacillus were the dominant genus after ensiling, and the undesirable microbes were inhibited in lower pH conditions, which resulted in lower BA, PA, and NH3-N production. In addition, strong bitterness, astringency, and sourness could reflect the tastes of alfalfa silage, which may be improved due to the changes in umami and sourness tastes with the amount of added M.

Overall, M addition improved the fermentation quality and changed the tastes of the alfalfa silage. Among all the treatments, the M3 group obtained the ideal pH value (below 4.5) and the best condition for long-term preservation.

## Figures and Tables

**Table 1:** Chemical composition of the alfalfa silage (*n* = 6).

Items	Treatment				*p* Value
	Control	M1	M2	M3	
DM (g/kg FM)	297.03 ± 1.74 ^c^	295.94 ± 0.49 ^c^	306.77 ± 0.73 ^b^	312.88 ± 0.75 ^a^	0.048
CP (g/kg DM)	245.75 ± 0.58	247.05 ± 0.32	242.04 ± 0.72	250.90 ± 0.69	0.149
WSC (g/kg DM)	31.71 ± 0.82 ^b^	28.52 ± 0.63 ^b^	37.19 ± 0.86 ^a^	32.19 ± 0.73 ^b^	<0.001
NDF (g/kg DM)	281.99 ± 0.99 ^a^	275.30 ± 1.46 ^a^	290.21 ± 2.27 ^a^	257.50 ± 1.18 ^b^	0.013
ADF (g/kg DM)	210.06 ± 0.52 ^a^	204.72 ± 1.04 ^a^	207.28 ± 0.75 ^a^	190.55 ± 0.90 ^b^	0.004

^a–c^ Means in the same row followed by different superscript letters are significant difference (*p* < 0.05). DM, dry matter; FM, fresh material; CP, crude protein; WSC, water-soluble carbohydrate; NDF, neutral detergent fiber; ADF, acid detergent fiber. M1, molasses addition at 1% of FM; M2, molasses addition at 2% of FM; M3, molasses addition at 3% of FM.

**Table 2:** The fermentation characteristics of the alfalfa silage (*n* = 6).

Items	Treatment				*p* Value
	Control	M1	M2	M3	
**pH**	5.16 ± 0.20 ^a^	4.84 ± 0.09 ^b^	4.63 ± 0.06 ^c^	4.48 ± 0.02 ^d^	<0.001
LA (g/kg DM)	67.21 ± 13.69 ^c^	80.53 ± 10.87 ^b^	81.59 ± 7.64 ^b^	96.90 ± 8.78 ^a^	<0.001
AA (g/kg DM)	21.18 ± 4.49 ^a^	20.55 ± 4.23 ^a^	15.81 ± 2.25 ^b^	14.31 ± 1.42 ^b^	0.006
PA (g/kg DM)	2.30 ± 0.73 ^a^	1.73 ± 1.06 ^a^	0.74 ± 0.35 ^b^	0.68 ± 0.26 ^b^	<0.001
BA (g/kg DM)	0.66 ± 0.60	0.46 ± 0.47	0.32 ± 0.23	0.08 ± 0.09	0.132
LA/AA	3.30 ± 0.88 ^c^	3.97 ± 0.36 ^bc^	5.21 ± 0.85 ^b^	6.84 ± 1.05 ^a^	<0.001
NH3-N (g/kg TN)	100.86 ± 27.90 ^a^	75.74 ± 4.60 ^b^	53.43 ± 4.69 ^c^	52.20 ± 3.73 ^c^	<0.001

^a–d^ Means in the same row followed by different superscript letters are significant difference (*p* < 0.05). DM, dry matter; LA, lactic acid; AA, acetic acid; PA, propionic acid; BA, butyric acid; TN, total nitrogen. M1, molasses addition at 1% of FM; M2, molasses addition at 2% of FM; M3, molasses addition at 3% of FM.

**Table 3:** Alpha diversity of bacterial diversity at the alfalfa silage (*n* = 6).

Group	Control	M1	M2	M3	*p* Value
Reads	109, 704	107, 709	109, 256	106, 625	/
OTUs	237.50 ± 58.70	240.60 ± 20.83	218.80 ± 68.36	210.17 ± 33.35	0.686
Shannon	2.58 ± 0.66 ^a^	2.65 ± 0.88 ^a^	1.59 ± 0.68 ^b^	1.75 ± 0.49 ^b^	0.037
Simpson	0.56 ± 0.15 ^a^	0.56 ± 0.18 ^a^	0.33 ± 0.19 ^b^	0.34 ± 0.11 ^b^	0.040
Chao	237.52 ± 58.69	240.65 ± 20.82	218.80 ± 68.36	210.17 ± 33.35	0.685
ACE	237.64 ± 58.65	240.80 ± 20.82	218.83 ± 68.41	210.28 ± 33.36	0.684
Coverage	0.99	0.99	0.99	0.99	0.107

^a,b^ Means in the same row followed by different superscript letters are significant difference (*p* < 0.05). M1, molasses addition at 1% of FM; M2, molasses addition at 2% of FM; M3, molasses addition at 3% of FM.

## Data Availability

Data is contained within the article.
